# Microsatellite Analysis in Multistage Carcinogenesis of Esophageal Squamous Cell Carcinoma from Chongqing in Southern China

**DOI:** 10.3390/ijms12117401

**Published:** 2011-10-28

**Authors:** Ming Liu, Feng Zhang, Shen Liu, Wen Zhao, Jing Zhu, Xiaoli Zhang

**Affiliations:** 1 Cardiothoracic Surgery Department, Affiliated South-West Hospital, Third Military Medical University, Chongqing 400038, China; E-Mail: liuming227@yahoo.com.cn; 2 Beijing Institute of Genomics of the Chinese Academy of Sciences, Beijing 100029, China; E-Mail: zhangf@big.ac.cn; 3 Department of Clinical Laboratory Sciences, Affiliated South-West Hospital, Third Military Medical University, Chongqing 400038, China; E-Mails: yashen1985@126.com (S.L.); zj6900@163.com (J.Z.); 4 Pathology Department, Affiliated South-West Hospital, Third Military Medical University, Chongqing 400038, China; E-Mail: xiaoli227@sina.com

**Keywords:** esophageal squamous cell carcinoma, microsatellite, loss of heterozygosity

## Abstract

In order to characterize the molecular events in the carcinogenesis of esophageal cancer and to identify biomarkers for the early detection of the disease, matched precancerous and cancerous tissues resected from 34 esophageal cancer patients in Chongqing of southern China were compared for the extent of loss of heterozygosity (LOH). Sixteen microsatellite markers on nine chromosome regions were used for the PCR-based LOH analysis. The overall frequency of LOH at the 16 microsatellite loci was significantly increased as the pathological status of the resection specimens changed from low-grade dysplasia (LGD) to high-grade dysplasia (HGD) and squamous cell carcinoma (SCC) (*P* < 0.001), indicating that tumorigenesis of the esophageal squamous epithelia is a progressive process involving accumulative changes of LOH. A total of eight markers showed LOH in the LGD samples, suggesting that these loci may be involved in the early-stage tumorigenesis of esophageal squamous cell carcinoma (ESCC) and that LOH analysis at these loci may help improve the early detection of this disease. In addition, heterozygosity was regained at four loci in the SCC samples of four patients compared with the HGD samples, suggesting the possibility of genetic heterogeneity in the tumorigenesis of esophageal cancer.

## 1. Introduction

Esophageal squamous cell carcinoma (ESCC) is a common type of malignant cancer in China, with 5-year survival rate less than 15%. Although radical esophagectomy is still the primary treatment for esophageal cancer, it is quite difficult to treat patients with distant or lymph-node metastasis. Therefore, identification of biomarkers that can improve the early diagnosis of esophageal cancer or precursor lesions will significantly reduce mortality in esophageal cancer patients. Similar to other solid tumors, chromosome loss is a common molecular defect in esophageal cancer. A study of chromosome loss in dysplasia and early-stage esophageal cancer will be helpful in the discovery of major tumor-associated genes and will lead to the understanding of the tumorigenesis of esophageal cancer. They will also be potential biomarkers for early detection of esophageal cancer. Although extensive genomic instability has been found in patients with esophageal cancer, the molecular alterations that are closely related to the different stages of tumorigenesis have not been fully understood. In order to further clarify the molecular alterations during the early-stage tumorigenesis of ESCC, and to identify potential biomarkers for early detection of the disease, we analyzed allelic losses at total of 16 microsatellite loci selected from chromosome regions 3p, 4p, 5q, 8p, 9p, 9q, 11p, 13q, and 17p in matched squamous dysplasia and squamous cancer tissues resected from 34 esophageal cancer patients in Chongqing of southern China.

## 2. Results

The overall frequencies of loss of heterozygosity (LOH) at the 16 microsatellite loci significantly increased as the pathological status of the resection samples changed (from low-grade dysplasia (LGD) to high-grade dysplasia (HGD) and to squamous cell carcinoma (SCC)) (Table 1). The overall frequency of LOH in the LGD samples (9.8%) was significantly lower than that in the HGD (48.6%) and SCC (58.5%) samples (*P* < 0.001). Although the LOH frequency at each locus showed an increasing trend with increasing disease severity, the difference was not statistically significant. Eight loci (D3S1597, D3S2452, D3S1285, D4S174, D5S2501, D9S125, D13S153, and D17S786) showed LOH in the informative LGD samples, and another eight loci were found to present LOH in the informative HGD specimens. All 16 loci were found to have LOH in the SCC samples. Furthermore, by comparing the occurrence of LOH in samples with different pathological statuses from the same patient, we found a regain of heterozygosity at loci D3S2452, D4S174, D9S125 and D17S261, in the SCC samples of four patients, respectively comparing to the HGD specimens which showed LOH at the corresponding loci.

## 3. Discussion

A total of 16 highly polymorphic microsatellite markers from nine chromosome regions with a high frequency of allelic loss in esophageal cancer were selected. Surgically resected squamous dysplasia and SCC samples from 34 esophageal cancer patients were subjected to LOH analysis at these loci. The results showed that the overall frequencies of LOH at the 16 microsatellite loci significantly increased as the pathological status of the resection specimens deteriorated (*i.e.*, from LGD to HGD and to SCC). These results indicated that tumorigenesis of esophageal squamous epithelia is a progressive process involving a series of molecular alterations. As the alterations accumulate to a certain degree, the cell morphology and behavior undergoes a radical change, leading to malignancy [1]. This finding was consistent with the studies for other tumor types, such as prostate cancer, colon cancer, and breast cancer [2–5]. Moreover, the results were also in accordance with those of a previous study showing that increasing grades of dysplasia were associated with an increased risk of developing ESCC [6].

Of 16 loci, we found eight loci to show LOH in low-grade dysplasia, indicating that LOH at these eight loci may be involved in the early-stage tumorigenesis of ESCC, and that these specific loci could be used as markers for screening of esophageal cancer. At present, some tumor-associated genes have been identified near these loci, such as *FHIT* (3p), *RASSF1A* (3p), *APC* (5q), *ANXI* (9q), *DEC1* (9q), *RB1* (13q), *BRCA2* (13q), *ING1* (13q), and *TP53* (17p). The expression of ANXI in chromosome 9q has been reported to be closely related to the tumor progression in patients with breast cancer and possibly plays an important role in the early stage of tumorigenesis [7]. Recently, study of this gene in esophageal cancer showed that its expression at protein level in cancer tissues is significantly lower than that in normal tissues [8]. Another candidate gene *DEC1* at chromosome 9q has been reported to be down-regulated in esophageal cancer, and transfection of the cDNA of *DEC1* could inhibit the proliferation of some cancer cells, suggesting that it may participate in the development of esophageal cancer [9]. The chromosome 13q region has a high frequency of allelic loss in esophageal cancer. However, the identified candidate genes within this region, such as *RB1*, *BRCA2*, and *ING1*, seldom show mutations in esophageal cancer, implying that other unknown tumor-suppressor genes within this region might participate in the tumorigenesis of this cancer [10]. The chromosome 17p region involving the *P53* locus also shows a high frequency of allelic loss in esophageal cancer. Studies of the *P53* gene in esophageal cancer have shown that LOH and mutations are the leading causes of its inactivation, which is in accordance with the “two-hit” model of tumorigenesis and indicates that *P53* is a major tumor suppressor in esophageal cancer [11].

In addition, eight other loci were found to show LOH in the HGD samples, indicating that LOH at these loci may be involved in the late stages of tumorigenesis (such as invasion and metastasis) in esophageal cancer.

This study also compared the occurrence of LOH in samples of different pathological statuses from the same patient. Interestingly, in four patients, LOH was found at some loci in the HGD samples, whereas heterozygosity was regained at the same loci in matched SCC samples. The regain of heterozygosity at some loci in tumor tissues indicated that the tumorigenesis of esophageal cancer may show genetic heterogeneity, *i.e.*, the HGD and SCC samples of the same patient may have been derived from different tumor-cell clones. Cells with different molecular defects may have distinct tumorigenesis processes, leading to different tumor-cell clones. A similar result has been found in adenocarcinoma of the esophagus; the molecular defects found in the severe dysplastic lesions were not detected in the invasive adenocarcinoma lesions of the same patient [12]. A study of patients with oral squamous cancer has also shown that most of the molecular alterations found in the precursor lesions were not revealed in the matched tumor tissues [13]. Similar observations have been reported in studies of other cancers such as breast cancer and prostate cancer [14,15].

Finally, it is worth mentioning that in some LGD samples of our subjects, there were already multiple loci to exhibit allelic loss, indicating that the earliest molecular event in esophageal cancer may occur in the histologically normal squamous epithelia. In a recent study on esophageal cancer, the researcher found that 19% of the histologically normal epithelia within the abnormal mucosal region (unstained with iodine) already showed LOH alterations, suggesting that the histologically normal epithelia adjacent to the tumor tissues may have early-stage LOH alterations, which might be necessary for the tumorigenesis of esophageal cancer [16]. Although at present, histological examination is still an effective method for the early detection of cancer, molecular tests such as LOH analysis of certain specific loci at the early stage can be a promising and more rational strategy for the early diagnosis or prediction of esophageal cancer.

## 4. Experimental Section

### 4.1. Sample Collection

The resection specimens from a total of 34 esophageal cancer patients were obtained from the Department of Thoracic Surgery, South-west Hospital, Chongqing, China. The patients included 23 men and 11 women with an average age of 58.6 years (44–74 years). All the patients were newly diagnosed with esophageal cancer, did not have a history of any other malignant cancer, and had not undergone any radiotherapy or chemotherapy. The patients were all Han Chinese who had lived in Chongqing for more than 20 years and did not have any direct kinship with each other. The resected samples were placed in liquid nitrogen immediately after the surgical removal and were preserved at −80 °C before use. Besides the cancerous foci, surrounding tissues were also collected from the 34 patients. The tissue samples were pathologically diagnosed with mild, medium, and severe dysplasia and SCC [17]. This study classified mild and medium dysplasia as low-grade dysplasia (LGD) and severe dysplasia as high-grade dysplasia (HGD). In addition, peripheral blood samples were collected before surgery. Informed consent had been obtained from all the study subjects in accordance with the standards established by the local institutional review boards.

### 4.2. Tissue Cell Acquisition by Using Microdissection

The frozen sections of squamous-cancer tissue and dysplastic tissue (5 μm) were stained with hematoxylin and eosin (HE) and subjected to microdissection under a 40× dissecting microscope to ensure that the proportion of tumor cells and dysplastic cells was higher than 80%, which would significantly reduce the rates of false positive or negative results during LOH analysis.

### 4.3. Extraction of Genomic DNA

Genomic DNA was extracted from the patients’ blood samples and tissues by using the DNeasy Blood and Tissue Kit (QIAGEN).

### 4.4. Loss of Heterozygosity (LOH) Analysis

A total of 16 microsatellite loci were chosen from the 3p, 4p, 5q, 8p, 9p, 9q, 11p, 13q, and 17p chromosome regions with a high frequency of allelic loss in esophageal cancer. A majority of these markers were dinucleotides and their maximum heterozygosity was more than 70%. Also the locus was confirmed as informative in our subjects. The resected samples and paired blood samples from the 34 esophageal cancer patients were analyzed for LOH at the 16 loci. Information about these markers and primer sequences is available on the Genome Database [18] and the NCBI genome database [19]. The primers were obtained as labeled primers with fluorescent dye at the 5-terminus (MWG Biotech, Ebersberg, Germany).

LOH analysis was performed as described in our previous paper [20]. In brief, the 5 μL volume of microsatellite DNA amplification included 40 ng of DNA template, 1× Buffer, 200 μM dNTPs, 250 nM microsatellite primer, 2.5 mM MgCl_2,_ and 0.25 U of Taq DNA polymerase. The PCR conditions were as follows: predenaturation at 94 °C for 3 min, followed by 30 cycles of 94 °C for 30 s→55~60 °C for 30 s→72 °C for 1 min, and a final elongation step at 72 °C for 4 min. PCR products were loaded on a fluorescent sequencer gel (ABI PRISM 377). The images were captured, converted, and analyzed by using the GeneScan software, and the size of each allele fragment was automatically calculated.

**Figure 1:**
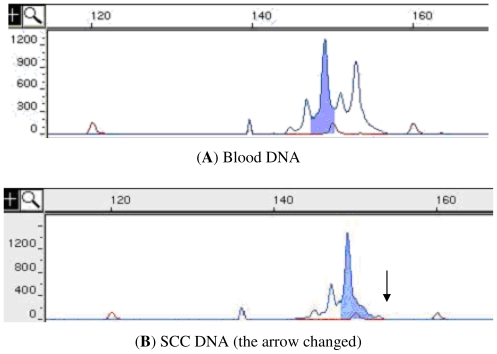

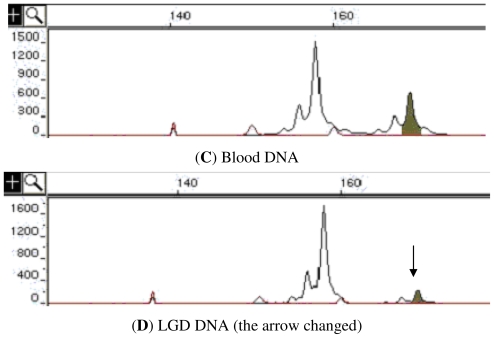
The X-coordinate represents the size of the fragment, and the Y-coordinate represents the fluorescence intensity. (**A**,**B**) Allelotyping results of the D3S2452 locus from the peripheral-blood samples and cancer-tissue samples of a patient. DNA from the peripheral-blood sample shows heterozygosity at the D3S2452 locus with fragments of 2 sizes—149 bp and 153 bp, whereas DNA from the tumor tissue only shows a single fragment—149 bp (arrowhead indicates the loss of a 153-bp fragment). These results indicate that LOH occurred at the D3S2452 locus in the cancer tissue. (**C**,**D**) Allelotyping results of the D4S174 locus from the peripheral blood and LGD-tissue samples of a patient. Both the peripheral blood and LGD samples comprise segments of 2 sizes—157 bp and 169 bp—at the D4S174 locus; the fluorescence intensity at the 169 bp segment (arrowhead) in the LGD sample was reduced by at least 30% of that in the peripheral-blood sample. The results highlight the presence of LOH at the D4S174 locus in the LGD sample. SCC: squamous cell carcinoma; LGD: low-grade dysplasia, including mild and medium dysplasia (in this study).

If the peripheral blood DNA of a patient showed heterozygosity at a certain locus, then the locus was marked as informative. The tissue sample of the patient was considered to show LOH if the DNA from the sample showed homozygosity at the informative locus or if the fluorescence intensity of one of the 2 alleles was less than 30% of that in the blood DNA of the patient (Figure 1). The frequency of LOH at each locus was defined as the total number of tissue samples with LOH at this locus/the total number of informative samples.

### 4.5. Statistical Analysis

The LOH frequencies at each locus between the different groups were compared by using the χ^2^ test, and a *P* value less than 0.05 was considered statistically significant.

## 5. Conclusions

The tumorigenesis of ESCC is a progressive process involving the accumulative alterations of LOH. The identification of eight loci already showing allelic loss in LGD suggests that they may be associated with the early-stage tumorigenesis of esophageal cancer, and could be used as molecular markers for early detection or prediction of this cancer.

## Figures and Tables

**Table 1:** The frequencies of loss of heterozygosity (LOH) at 16 microsatellite loci in the squamous dysplastic tissues and esophageal squamous cell carcinoma (ESCC) tissues.

Marker	Location	LGD a sample (%)	HGD a sample (%)	SCC a sample (%)
3S1597	3p25	4/21 (19)	12/22 (54.5)	18/22 (81.8)
D3S2452	3p21-p14	7/25 (28)	20/25 (80)	18/25 (72)
D3S1285	3p14	3/19 (15.8)	14/21 (66.7)	16/21 (76.2)
D4S174	4p14-p13	2/27 (7.4)	7/27 (25.9)	12/27 (44.4)
D5S409	5q14-q15	0/16 (0)	7/16 (43.7)	8/16 (50)
D5S2501	5q21-q23.3	1/13 (7.7)	7/13 (53.8)	7/13 (53.8)
D8S261	8p22-p21.3	0/18 (0)	5/18 (27.8)	6/18 (33.3)
D9S157	9p23-p22	0/23 (0)	16/23 (69.5)	15/23 (65.2)
D9S111	9q12-q21.1	0/25 (0)	9/25 (36)	15/25 (60)
D9S125	9q34-q34	9/27 (33.3)	21/27 (77.8)	23/27 (85.2)
D11S1338	11p15.5	0/29 (0)	10/29 (34.5)	11/29 (37.9)
D13S175	13q13	0/22 (0)	10/23 (43.5)	12/23 (52.2)
D13S153	13q14.2	4/24 (16.7)	10/24 (41.7)	13/24 (54.2)
D13S173	13q32-q34	0/24 (0)	5/24 (20.8)	11/24 (45.8)
D17S786	17p13.1	5/23 (21.7)	13/23 (56.5)	16/23 (69.5)
D17S261	17p11.2	0/22 (0)	10/22 (45.4)	11/22 (50)
Total b		35/358 (9.8)	176/362 (48.6)	212/362 (58.5)

a LGD: low-grade dysplasia, including mild and medium dysplasia (in this study); HGD: high-grade dysplasia, *i.e.*, severe dysplasia; SCC: squamous cell carcinoma;

b If *P* < 0.001, then the difference is statistically significant.
